# Mucosal-associated invariant T cells in hematological malignancies: Current knowledge, pending questions

**DOI:** 10.3389/fimmu.2023.1160943

**Published:** 2023-03-20

**Authors:** Emmanuel Treiner

**Affiliations:** ^1^ Infinity, Inserm UMR1291, Toulouse, France; ^2^ University Toulouse 3, Toulouse, France; ^3^ Laboratory of Immunology, Toulouse University Hospital, Toulouse, France

**Keywords:** MAIT cells, MR1, tumor immunology, immunotherapy, leukemia

## Abstract

Non-classical HLA restricted T cell subsets such as γδ T and NK-T cells are showing promises for immune-based therapy of hematological malignancies. Mucosal-Associated Invariant T cells (MAIT) belong to this family of innate-like T cell subsets and are the focus of many studies on infectious diseases, owing to their unusual recognition of bacterial/fungal metabolites. Their ability to produce type 1 cytokines (IFNγ, TNFα) as well as cytotoxic effector molecules endows them with potential anti-tumor functions. However, their contribution to tumor surveillance in solid cancers is unclear, and only few studies have specifically focused on MAIT cells in blood cancers. In this review, we wish to recapitulate our current knowledge on MAIT cells biology in hematological neoplasms, at diagnosis and/or during treatment, as well as tentative approaches to target them as therapeutic tools. We also wish to take this opportunity to briefly elaborate on what we think are important question to address in this field, as well as potential limitations to overcome in order to make MAIT cells the basis of future, novel therapies for hematological cancers.

Since the initial discovery of unconventional T cell subsets in the 1980s, there has been a large interest in the possibility of harnessing the power of γδ T cells, CD1-restricted, and more recently, MR1-restricted T cells (MR1T), to treat or prevent diverse pathologies such as autoimmune diseases, infections or cancer. Whereas a great deal of work has been conducted with the two former cell populations, leading to the development of start-up companies and several clinical trials, the field of MR1T cells and their potential as therapeutic candidates is still in its infancy.

Mucosal-Associated Invariant T cells represent the vast majority of T cells restricted to the non-classical, MHC-class Ib molecule, MHC-Related 1 (MR1). Their innate-like recognition of microbial metabolites, their potent anti-microbial functions, and their localization at mucosal surfaces, have drawn tremendous interest in the context of infections (1–3). In agreement with MAIT cells being positioned close to mucosal surfaces, several studies have logically focused first on colorectal cancer, followed by analyses of other epithelial cancers (4, 5). MAIT cells functions clearly are relevant to tumor immunotherapy, including direct production of IFNγ and cytotoxic molecules (perforin, granzymes, granulysin) (6, 7), but also the capacity to interact with dendritic cells in a way that would promote their maturation and subsequent priming of anti-tumor T cell responses (8). Surprisingly, the presence of MAIT cells in the tumors seem to be detrimental in several studies (3, 9). Whether this is the consequence of dysfunctional intra-tumoral MAIT cells (4), a functional shift to pro-tumoral functions, or any other mechanism(s) (10), remain to be explored. Nonetheless, MAIT cells hold promises for immune therapy of cancer, provided an important endeavor is carried out to deeply analyze their pro- versus anti-tumor functions in animal models and in humans.

Most studies on MAIT cells focused on solid cancers, but very little is known about MAIT cells and their role, if any, in the pathophysiology and/or treatment of hematologic malignancies. In this review, we will first describe the current knowledge about the behavior of MAIT cells in patients with hematologic malignancies. We will review published work as well as future avenues of research on therapeutic interventions, either directed at manipulating MAIT cells (e.g. CAR-MAIT), or their implication in response to other types of therapeutics (such as checkpoint blockade). Finally, we will raise issues that we think are important when considering possible interventions implicating this peculiar T cell subset.

## MAIT cells in hematologic malignancies

The first study describing MAIT cells in patients with hematologic malignancies focused on multiple myeloma (MM) (11). The authors observed a reduced frequency of circulating MAIT cells in newly diagnosed patients, that paralleled the decreased levels of another major innate-like T cell subsets, CD1d-restricted NK-T cells. MAIT cells were also reduced in bone marrow (BM), arguing against a redistribution of these cells in the tumor microenvironment. MAIT cells showed an altered capacity to produce type 1 cytokines (IFNγ, TNFα) upon mitogenic stimulation. Further, *in vitro* activated NK-T cells (with α-galactosyl ceramide) trans-activated MAIT cells, but this capacity was reduced in MM patients. Interestingly, MAIT cells expressed higher levels of PD-1 in MM patients compared with healthy donors, and antibody-mediated blockade of PD-1 partially restored MAIT cells transactivation by NK-T cells.

Another study (12) rapidly confirmed these early results, i.e. decreased numbers and functions of circulating MAIT cells in MM patients at diagnosis. Again, *in vitro* there was no evidence for an accumulation of MAIT cells in the bone marrow (BM), suggesting that the lower numbers were not due to redistribution, but rather to aging, and/or an increased apoptosis and/or a decreased survival. Intriguingly, the IFNγ secretion was normal in refractory or relapsed patients, as compared to untreated, newly diagnosed individuals. Finally, MM cell lines expressed MR1 and were targets for MAIT cell killing upon addition of the specific MAIT ligand 5-(2-oxopropylideneamino)-6-d-ribitylaminouracil (5-OP-RU). These two studies were important in showing for the first time alterations of MAIT cells homeostasis and functions in the context of a specific type of blood cancer, but also that they displayed significant anti-tumor capacity in this context.

In a recently published work, we prospectively monitored MAIT cells in a cohort of 216 patients with newly diagnosed acute myeloid leukemia (AML). As in MM, we observed a decline in blood MAIT cells frequency and concentration (13). The decreased MAIT cells frequency was inversely correlated to activation, as measured with HLA-DR expression. Interestingly, MAIT cells dynamics was also associated with the cytogenetic profile and the molecular subtypes of AML. Thus, blood MAIT cells were lower in patients with an adverse cytogenetic profile. Further, a reduced number of patients harbored activated MAIT cells within the favorable prognosis group, as compared with the adverse or intermediate groups. This could lead to the hypothesis that MAIT cells are activated in subsets of AML patients, and this activation would be associated with an adverse prognosis. We also analyzed MAIT cells in patients with an intermediate cytogenetic profile, in association with the presence or absence of specific gene mutations. We found, in contrast, higher blood MAIT cells frequencies in patients harboring FLT3-ITD mutations (associated with a pejorative prognosis), as well as with IDH1/2 mutations. Thus, the interplay between molecular events linked to tumor progression, and MAIT cells behavior in AML is probably more complex and dependent upon other unexplored parameters.

Interestingly, a recent article (14) described the clonal expansion of MAIT cells in the BM of patients with a specific subtype of AML (NK AML M4/M5). Intriguingly, the only patient not achieving complete remission showed a larger MAIT cell infiltration and expression of a specific gene transcription signature. This is the first and only study to date showing BM infiltration by MAIT cells. Further, this work and others suggest that activation of MAIT cells in AML could result in infiltration in the tumor microenvironment, with possible negative impact on disease course. Alternatively, MAIT cells activation could induce MAIT cells apoptosis, explaining the decreased frequency of circulating cells.

Finally, patients with chronic lymphocytic leukemia (CLL) harbored decreased numbers of circulating CD8+CD26hi T cells (15). It was already shown that MAIT cells express high levels of CD26 (13, 16), and the authors confirmed the predominance of MAIT cells within the CD8+CD26hi subset. This study therefore suggest that CLL may also impact MAIT cells physiology, although this requires further confirmation.

Altogether, MAIT cells numbers and/or functions are altered in three different, unrelated types of hematologic cancer. However, whether similar or different mechanisms account for these alterations remains to be established.

### Impact of – and on – therapy of hematological neoplasms

#### Hematopoietic stem cell transplantation

MAIT cells were shown to be quite resistant to chemotherapy, and reconstitute rapidly after autologous HSCT following myeloablative regimen for treatment of non-hodgkin lymphoma (NHL) or MM (17). Further, pre-treatment MAIT levels was associated with better early clinical outcome, with patients displaying less post-transplant infections (as assessed by indirect markers such as CRP levels, or antibiotics uptake). However, other cell types were not analyzed in that study, precluding the assessment of causality in that correlation.

In an allogenic setting, after either myelo- or non-myeloablative conditioning, MAIT cells levels rose rapidly, but failed to normalize to pre-transplant levels (18, 19). Of note, patients in this latter study, diagnosed with various malignancies (NHL, AML, ALL, MDS…) had low pre-transplant circulating MAIT cell levels, confirming that this cell subset is profoundly affected in different sorts of blood malignancies. Importantly, early increase came from graft-transferred MAIT cells, and proliferation was associated with an increased abundance of Blautia *spp* in the gut microbiota. This important study questioned the resistance of MAIT cells to chemotherapy, as reconstitution mostly arose from graft-derived MAIT. Further, MAIT cells displayed regulatory activity against proliferating conventional T cells, and therefore, a Graft-versus-host disease (GVHd)-protecting effect. Along the same line, another study showed a correlation between MAIT cell numbers post-HSCT and the occurrence of GVHd, where a threshold of 0.48/mm3 could be calculated as an independent risk factor for the risk of GVHd (20). These studies suggest that MAIT cells are protective against GVHd, and that including MAIT cell numbers in the selection of grafts, and/or mobilizing MAIT cells within the graft, could have a positive outcome. It must be noted, however, that not all studies found a positive effect of MAIT cells with respect to GVHd (18).

MAIT cells respond to bacterial ligands from the microbiota, and the diverse studies showed a benefit for microbial diversity within the microbiota with favorable outcome after allo-HSCT. A recent work (21) demonstrated a link between these different events, where a more diverse microbiota improved the reconstitution of MAIT and Vδ2 unconventional T cells, which in turn contributed to a decrease in acute GVHd severity and improved survival. MAIT levels were associated with an abundance of riboflavin producing bacteria, belonging to the phylum *Bacteroidetes*. The mechanisms underlying the beneficial effect of increased MAIT cells reconstitution are not known, but the authors described the activation status of these cells, with high cytotoxic and Th1/Tc1 functional profile. All this was also concordant with another study demonstrating an inverse correlation between the number of MAIT cells in graft and the risk of acute intestinal GVHd, and the activation of MAIT cells by riboflavin-producing bacteria post-HSCT (22). Of note, MAIT cells post-HSCT expressed CXCR4, which can induce attraction of hematopoietic cells to the BM in response to CXCL12 production by stromal cells. Again, only one study claimed an association of higher MAIT in PB grafts with higher risk of aGVHD (23).

The role of MAIT cells in GVHd was addressed in a murine model of allo-HSCT (24). MAIT cells were found in target organs of GVHd such as liver, lung and colon, and provided protection against GVHd upon adoptive transfer. Mechanistically, they inhibited allogenic CD4 T cell responses, possibly through dampening of allogenic antigens presentation by LN-DC, specifically in the colon. This effect was dependent upon IL-17 production, and associated with the shaping of the gut microbiota. Importantly, in contrast with the human data, the regulatory functions of MAIT cells in this model were confined to recipient MAIT cells, which resisted irradiation, and not transferred donor MAIT from the grafts.

#### Chemotherapy

Most current treatments of hematologic malignancies unfortunately do not spare lymphocytes, resulting in lymphopenia and altered leucocyte functions in a number of patients. Whether or not MAIT cells are sensitive to these treatments has been only questioned in a limited number of studies.

MAIT cells express higher levels of the multi-drug resistance protein-1 (MDR1, or P-Glycoprotein) than other CD8+ T cell subsets (25, 26) . MDR1 is a drug efflux pump, implicated in cancer resistance to multiple chemotherapies, such as anthracyclins, with consequences on treatment efficacy of acute leukemia (27–29). Indeed, in AML patients treated with the anthracyclin daunorubicin, MAIT cells resist daunorubicin-induced apoptosis *in vitro* and *in vivo* (13, 26, 30).

On the other hand, cyclophosphamide, an alkylating agent used for conditioning before HSCT, has a very strong depletion effect on MAIT cells (19). However, cyclophosphamide is also used to treat lymphoma, MM and ALL, where this MAIT-depleting effect might be deleterious. Similarly, the MM treatment lenalidomide had a profound inhibitory effect on MAIT cells (12).

In a search for the identification of new MR1 ligands, Keller et al. screened a number of compounds, including FDA-approved drugs (31). *In silico* modeling was followed by *in vitro* experiments looking for compounds that upregulated MR1 on the surface of C1R cells transfected with MR1, and/or activated Jurkat cells expressing a MAIT-derived TCR. Among the compounds able to activate MAIT cells, degradation products of methotrexate, and mercaptopurin, were found to be weak agonists of MAIT cells activation. Both drugs are chemotherapeutics agents used in various hematologic malignancies, which raises the prospect that their use may induce some kind of MAIT cells activation *in vivo*, and, possibly, participate in their activity.

A few studies claimed that MAIT cells may respond to immune checkpoint blockade (ICB) *in vitro*. As already mentioned, MAIT cells from MM patients express high PD-1 and show increased functionality upon PD-1 blocking *in vitro*; similar results were documented for prostate cancer patients (11, 32). Interestingly, exhausted MAIT cells with a PD-1^high^Tim3^+^CD39^+^ phenotype accumulate in colon tumors, and their polyfunctionality may be improved by the PD-1 antagonist pembrolizumab (33). Finally, a recent report suggested that MAIT cells may respond to PD-1 blockade *in vivo* in a small cohort of metastatic melanoma patients (34). In ICB responders, MAIT cells showed direct evidences of activation and proliferation along the course of treatment. Further, higher baseline MAIT cells frequencies was associated with increased probability of response, suggesting their use as biomarkers of clinical response to ICB. Whether or not MAIT cells respond directly to ICB, or represent markers of immune competence in this context should be investigated in the future.

Altogether, the available data suggest that the differential usage of chemotherapeutic agents and/or immunotherapeutic modulators have an impact on MAIT cells, which could be taken into account in future therapeutic designs. However, the response of MAIT cells to a number of current therapeutics have not, and should be addressed in the future.

In sum, our current knowledge on MAIT cells in hematological malignancies is very limited, but the few reports and data available should foster an endeavor to better characterize the relationship between MAIT cells, tumor blasts and the environment. In line with this, we believe there are many questions to be addressed before any engaging into any kind of MAIT cells-based therapy of blood cancers.

### Do MAIT cells display anti-tumor functions?

The anti-tumoral functions of MAIT cells were assessed *in vivo* in several models of transplanted tumors, where B16F10 melanoma cells were injected either in the liver, the blood, or subcutaneously. MAIT cells activated with MR1 ligands (5-OP-RU) combined with CpG, a TLR9 ligand, infiltrated the tumor site, decreased tumor growth and increased survival. This anti-tumor effect was abolished in MR1^-/-^ mice, but it did not require MR1 expression on the tumor cells, pointing to an indirect role for MAIT cells, possibly through activation of other type(s) of effector cells (35) Indeed, another study showed in a lung tumor model a tumor-controlling function of activated MAIT cells; mechanistically, anti-tumor functions were dependent upon MR1 expression by tumor cells, and directly involved NK cells. Thus, it was suggested that MAIT cell activation induced NK cells to kill tumors. Oppositely, MAIT cells deficiency resulted in an enhanced control of tumors by NK cells. Thus, MAIT cells seemingly play a dual and opposite role on tumor control in these models, both through the regulation of NK cell functions. This could reconcile other data obtained in solid cancers, where MAIT seemed to play either anti-tumor or tumor-promoting functions (9, 36). How this double-edge sword function plays out in the context of leukemias and lymphomas is not known and should be properly addressed.

### Do MAIT cells recognize hematopoietic tumor cells?

Although MAIT cells display putative anti-tumoral functions, such as cytotoxicity and IFNγ production, it is unclear whether they may specifically recognize and target tumor cells. It has been shown in several *in vitro* experiments that cell lines of various origins do not spontaneously activate MAIT cells, but do so in the presence of activating MR1 ligands.

As already mentioned, MM cell lines incubated with 5-OP-RU are targets for MAIT cells-mediated killing (12). However, whether primary blasts in MM and other diseases globally retain MR1 expression is not known. Further, killing required addition of MR1 ligands, arguing against spontaneous anti-tumor activity in this context. Another study showed *in vitro* lymphokine-activated Killer activity by MAIT cells against the leukemic K562 cell line (5), though the MR1-dependence of this phenomenon was not addressed. Intriguingly, it appears that MR1 indeed may present endogenous and tumor-associated antigens to non-MAIT MR1-restricted T cells (37, 38). In the later study, the addition of MAIT-specific MR1 ligands, derived from riboflavin metabolism, actually reduced the activation of these anti-tumoral MR1-restricted T cells, suggesting the possibility that MAIT cells activation could actually antagonize MR1-restricted anti-tumor functions.

MHC class I downregulation is common in cancer, including hematologic malignancies and induces escape from CD8 T cells killing (39). It is most often the consequence of β2m mutations impairing its expression. Although MR1 expression on blast cells has been seldom studies, it is predicted to be impaired in cells losing β2m expression. This may be an issue when considering any strategy that would require cognate TCR-dependent activation of MAIT cells by tumor cells.

## Harnessing MAIT cells for therapy of hematologic malignancies

### car-mait

CAR (Chimeric Antigen Receptors)-T cells constitute a novel and expanding approach to treat cancers. Patients’ T cells are transduced with a construct encoding a chimeric molecule made of an anti-antigen antibody-like extracellular domain fused to the transducing domain of the CD3ζ chain, and other molecules (such as CD28 or 4-1BB (40)). There, T cells are endowed with Ig- like reactivity against tumors, but retain their specific functions such as cytotoxicity and cytokine secretion. To date, regulatory agencies have approved the use of CD19-CAR-T and BCMA-CAR-T for treatment of refractory ALL and MM, respectively.

Despite showing an impressive efficacy, CAR-T cells show important limitations. First, bulk T cells are transduced, including CD4+, CD8+ T cells with undefined functions. Second, CAR-T cells are generated from autologous T cells, because the use of allogenic T cells expressing endogenous TCRs would create a risk of GVHd. Thus, the overall process is performed through a complex procedure in centralized accredited laboratories, with important delays and even failure to successfully express the desired construct in the patient’s own cells (41).

In this context, MAIT cells represent attractive candidates for “off-the-shelf” CAR-T cell therapy, since they represent a homogenous population devoid of alloreactive potential (42, 43).

Indeed, Dogan et al. (44) recently demonstrated the feasibility of engineering MAIT cells as a platform for CAR-T cell therapy. After transduction with either a CD19- or a Her2neu – CAR construct, MAIT cells were expanded *in vitro* and demonstrated high cytotoxicity against cell lines expressing the respective cognate antigens. Although preliminary, this is an encouraging proof-of-concept study, which will require further validation in animal models, not only to validate the efficiency of CAR-MAIT. Indeed, CAR-T cell therapy is accompanied by frequent, sometimes severe, toxic reactions, which is a significant limit to its wide usage; whether CAR-MAIT would show similar toxicity is important to assess.

### Induced pluripotent stem cells

iPSC represent another novel technology that could widen the therapeutic arsenal against cancer. The ability to derive tumor-fighting lymphocytes through reprogramming can overcome various roadblocks in adoptive transfer therapy, such as immune exhaustion among others (45). Although most efforts seem to focus on iPSC-derived NK cells in the context of tumor immunology, donor-unrestricted T cells represent another attractive target. Indeed, Wakao et al. demonstrated in a series of papers the possibility of reprogramming both murine and human MAIT cells through iPSC technology (46, 47). iPSC-derived murine MAIT cells showed potent anti-tumoral functions upon adoptive transfer in a lung metastasis model, in part through an enhancement of NK cells-mediated cytolysis. CAR-MAIT could also be generated from iPSC-derived MAIT cells. Thus, this technology represent another way MAIT cells could be manipulated in an off-the-shelf product amenable to the clinics.

### MAIT-BiTEs

BiTEs (Bi-specific T cell engagers) are engineered monoclonal antibodies where each arm of the antibody binds to a specific surface molecule, bringing together a target (tumor) cell with a T cell cytolytic effector, and activating the latter. For instance, Blinatumomab is a CD3/CD19 BiTE approved for treatment of B-ALL. A growing number of BiTEs are currently in development and in clinical trials, such as CD123/CD3 or CD33/CD3 BiTEs for AML, among others (48). Given the aforementioned features of MAIT cells, directly targeting this subset could represent an alternative to non-specific T cell engagement. Indeed, a recent report described the production of a BiTE engaging the TCR chain TRAV1-2 (expressed by the vast majority of MAIT cells) and a Tumor-Associated Antigen, PD-L1 (49). The authors showed effective targeting of various cancer cell lines expressing PD-L1 and MAIT cells activation. This activation was poly-functional and induced the lysis of cell lines. This work represent the first proof-of-concept that specifically engaging MAIT cells may represent an option to treat malignancies, and should prompt further investigations in *in vivo* models.

### Cytokine therapy

Cytokine-based therapy has gained new attention in the field of tumor immunology, thanks to the development of strategies to overcome limitations pertaining to pharmacodynamics and toxicities (50). Several cytokines (IL-12, IL-15, IL-18, type 1 IFN) can either directly activate MAIT cells, and/or co-stimulate them (with TCR/CD3-stimulation) to engage in specific effector programs. Further, some of them may even help reverse MAIT cells dysfunctions, as shown for IL-7 in chronic HIV infection. Thus, specifically providing these cytokines to MAIT cells could be helpful, or even necessary in the context of hematological malignancies.

### MAIT cells as adjuvants for vaccine immunology

Once at the forefront of the research on immune-based therapy of cancer, therapeutic cancer vaccine studies have been shadowed by the successes of antibody- and cell-based therapies, ICB and CAR-T cells. However, these treatments do not represent a universal therapy to cancer, at least in the near future, due to important toxicities and/or lack/loss of response in many patients. This leads some experts to claim that the development of efficient cancer vaccines is still a relevant approach, at least for subsets of patients (51). In different models of infection, providing activating MAIT cells together with the administration of specific vaccines shows a significant adjuvant effect, i.e. increases vaccine immunogenicity (52–54). Cancer vaccines often lack immunogenicity and therefore could possibly benefit from this approach, as already shown for NKT cells (55–57).

## Problems to overcome, questions to address

### MAIT cell exhaustion

Healthy MAIT cells feature interesting and potent effector functions relevant to immune-mediated anti-cancer therapy. Unfortunately, akin to other T cell subsets, MAIT cells seem to be the subjected to various dysfunctional states, described in patients with various chronic diseases. Thus, MAIT cells can show increased expression of cell-surface co-inhibitory receptors (coIR), such as PD-1, CTLA-4, TIM-3 and/or CD39. First described in HIV infection (58) and HIV/TB coinfection (59), similar findings were found in chronic viral hepatitis (60–63), autoimmune diseases (64, 65), or solid cancers (9, 32, 33, 66). This increased level of coIR was often accompanied by stigmata of chronic activation, decreased circulating numbers, and decreased responsiveness. Further, this exhausted phenotype was persistant even after effective anti-viral therapy in HCV or HIV –treated patients (58, 67) . *In vitro*, repeated exposure to inflammatory cytokines induced sustained CTLA4 expression on MAIT cells, as a possible safeguard mechanism against immune pathology (68).

Thus, chronic stimulation, either through cognate interactions or inflammatory cytokines (65), seem to drive a dysfunctional state similar to exhaustion in MAIT cells, akin to conventional T cells. Further, it was shown that chronic stimulation also leads to IL13 and IL5 production alongside TNF and IFN (69). Type 2 cytokines may be detrimental for anti-tumor activity.

No study to date has studied MAIT cells exhaustion in the context of hematologic malignancies. However, as already mentioned, MAIT cells are numerically deficient in patients with MM and AML (12, 13); the reason for this is still obscure but one possibility would be that MAIT suffer from increased apoptosis (15). This would represent some kind of immune escape as well.

### MAIT cells plasticity

The first reports analyzing MAIT cells functions *in vitro* showed these cells to be type 1/17 cytokines producers (25), as well as cytotoxic effectors against bacterially infected cells (70). Surprisingly, although MAIT cells are mostly effector/memory cells, their effector functions seemed to be difficult to engage, requiring co-stimulation by inflammatory cytokines such as IL-12, IL-15, IL18 or type 1 IFN, including in *in vivo* models (71–73). As MAIT-specific ligands like 5-OP-RU can be derived from non-pathogenic microbes, such as commensals, this was interpreted as the existence of a sail-safe mechanism to limit inappropriate MAIT cells activation in the absence of danger (inflammatory) signals. In parallel, inflammatory cytokines could induce at least some effector functions in MAIT cells, in the absence of TCR triggering (74). More recently, however, several groups have highlighted the ability of MAIT cells to engage in different effector programs, according to the integration of activating stimuli (75, 76). Thus, beyond their pro-inflammatory, anti-infectious program, MAIT cells can turn into tissue-repair effectors, through the production of amphiregulin, among others mediators (77, 78).

MAIT cells engaging in this function could be ineffective or even detrimental to tumor immunity. Therefore, a perfect understanding of the factors controlling MAIT cells effector programs and their stability *in vivo* is required before any attempt to manipulate them in a therapeutic fashion.

### Aging

The dynamics of circulating MAIT cells numbers over time have been invariably shown to exhibit an asymetric bell-shape, with an increase over the first years of life (79), reaching maximum levels between the second and fourth decades, followed by a steady decline, whereby aged people display significantly fewer circulating MAIT (80–82). Their functional status as measured by intracellular expression of cytotoxic molecules, and mitogen-induced type 1 cytokines secretion, seem unchanged (82), but IL-4 production may be increased (80) suggesting a possible functional switch. As hematologic malignancies are more frequent in aged subjects, MAIT cells in these patients may be intrinsically less potent and more difficult to harness. Whether or not the decreased age-associated MAIT cells frequency somewhat contributes to the emergence of malignancies remains to be explored.

### Traffic to the tumoral microenvironment

Effector cytotoxic lymphocytes need to infiltrate tumors in order to get in close contact with, and kill, tumor cells. A number of hematologic malignancies, such as acute leukemias or multiple myeloma, originate from the bone marrow. If and how MAIT cells migrate to the BM remains underexplored. In MM or AML patients, MAIT cells were found in the BM of patients, but with no evidence of specific recruitment (12, 13) . It has been proposed that BM T cells originate from the blood and permanently recirculate through it (83), contributing to recall responses. Further, *in vitro* expanded BM-infiltrating lymphocytes display anti-tumoral functions after adoptive transfer in MM patients (84). Retention in the BM is dependent upon the expression of the VLA-4 integrin in murine CD8 T cells (85). MAIT cell activation through their TCR in combination with IL-18 was shown to upregulate VLA4 expression (86). Although BM CD8 T cells are mostly of the CM phenotype, whereas MAIT cells display more often an EM phenotype, this suggests the possibility that MAIT infiltrate the bone marrow in certain situations. CXCR4 is another important molecular cue in entry into the BM (87). MAIT cells also express CXCR4 (88, 89).

#### Immunosuppressive tumoral microenvironment

Tumor escape from the immune system frequently involves the formation of an immunosuppressive microenvironment, acting through multiple mechanisms to dampen immune effectors. This includes over-expression of immune checkpoint ligands (such as PD-L1 or Gal-9) by blasts themselves, but also the presence of various regulatory cells, such as CD4+ regulatory T cells (Tregs), Myeloid-Derived Suppressive Cells (MDSC), or tumor-associated macrophages (TAM), which inhibit the immune response through a number of mechanisms such as production of Indole-amine 2,3 dioxygenase (IDO), TGFbeta, arginase, IL-10, among many other immunosuppressive factors (90, 91). How these regulatory mechanisms affects MAIT cell functions is not known.

## Concluding remarks

The specifics of MAIT cells endow them with interesting features for future therapeutic interventions in cancer. In the context of hematological clonal disorders, very little is known about the possible anti-tumor functions of MAIT cells, but their possible regulatory role protecting against GVHd is promising. We have also tried in this manuscript to pinpoint several major issues pertaining to possible therapeutic manipulation; this was in no way meant to be exhaustive, as many other parameters should be taken into account, such as the interplay between MAIT cells and the microbiota. Thus, there is a need for increasing our research efforts to deepen our understanding of MAIT cells biology and implication in these disorders, which could bring novel approaches to treatment.

## Author contributions

The author confirms being the sole contributor of this work and has approved it for publication.

## References

[1] Salio M. Unconventional MAIT cell responses to bacterial infections. Semin Immunol (2022) 61-64:101663. doi: 10.1016/j.smim.2022.101663 36306661

[2] Sundstrom P Ahlmanner F Akeus P Sundquist M Alsen S Yrlid U. Human mucosa-associated invariant T cells accumulate in colon adenocarcinomas but produce reduced amounts of IFN-gamma. J Immunol (2015) 195(7):3472–81. doi: 10.4049/jimmunol.1500258 26297765

[3] Zabijak L Attencourt C Guignant C Chatelain D Marcelo P Marolleau JP. Increased tumor infiltration by mucosal-associated invariant T cells correlates with poor survival in colorectal cancer patients. Cancer Immunol Immunother (2015) 64(12):1601–8. doi: 10.1007/s00262-015-1764-7 PMC11028701 26497850

[4] Shaler CR Tun-Abraham ME Skaro AI Khazaie K Corbett AJ Mele T. Mucosa-associated invariant T cells infiltrate hepatic metastases in patients with colorectal carcinoma but are rendered dysfunctional within and adjacent to tumor microenvironment. Cancer Immunol Immunother (2017) 66(12):1563–75. doi: 10.1007/s00262-017-2050-7 PMC11029177 28798979

[5] Won EJ Ju JK Cho YN Jin HM Park KJ Kim TJ. Clinical relevance of circulating mucosal-associated invariant T cell levels and their anti-cancer activity in patients with mucosal-associated cancer. Oncotarget (2016) 7(46):76274–90. doi: 10.18632/oncotarget.11187 PMC5342813 27517754

[6] Mori L Lepore M De Libero G. The immunology of CD1- and MR1-restricted T cells. Annu Rev Immunol (2016) 34:479–510. doi: 10.1146/annurev-immunol-032414-112008 26927205

[7] Rudak PT Choi J Haeryfar SMM. MAIT cell-mediated cytotoxicity: Roles in host defense and therapeutic potentials in infectious diseases and cancer. J Leukoc Biol (2018) 104(3):473–86. doi: 10.1002/JLB.4RI0118-023R 29668066

[8] Salio M Gasser O Gonzalez-Lopez C Martens A Veerapen N Gileadi U. Activation of human mucosal-associated invariant T cells induces CD40L-dependent maturation of monocyte-derived and primary dendritic cells. J Immunol (2017) 199(8):2631–8. doi: 10.4049/jimmunol.1700615 PMC5632842 28877992

[9] Duan M Goswami S Shi JY Wu LJ Wang XY Ma JQ. Activated and exhausted MAIT cells foster disease progression and indicate poor outcome in hepatocellular carcinoma. Clin Cancer Res (2019) 25(11):3304–16. doi: 10.1158/1078-0432.CCR-18-3040 30723143

[10] Yan J Allen S McDonald E Das I Mak JYW Liu L. MAIT cells promote tumor initiation, growth, and metastases *via* tumor MR1. Cancer Discov (2020) 10(1):124–41. doi: 10.1158/2159-8290.CD-19-0569 31826876

[11] Favreau M Venken K Faict S Maes K De Veirman K De Bruyne E. Both mucosal-associated invariant and natural killer T-cell deficiency in multiple myeloma can be countered by PD-1 inhibition. Haematologica (2017) 102(7):e266–e70. doi: 10.3324/haematol.2017.163758 PMC5566052 28385777

[12] Gherardin NA Loh L Admojo L Davenport AJ Richardson K Rogers A. Enumeration, functional responses and cytotoxic capacity of MAIT cells in newly diagnosed and relapsed multiple myeloma. Sci Rep (2018) 8(1):4159. doi: 10.1038/s41598-018-22130-1 29515123 PMC5841305

[13] Comont T Nicolau-Travers ML Bertoli S Recher C Vergez F Treiner E. MAIT cells numbers and frequencies in patients with acute myeloid leukemia at diagnosis: Association with cytogenetic profile and gene mutations. Cancer Immunol Immunother (2022) 71(4):875–87. doi: 10.1007/s00262-021-03037-9 PMC10991316 34477901

[14] Wu W Liang X Li H Huang X Wan C Xie Q. Landscape of T cells in NK-AML(M4/M5) revealed by single-cell sequencing. J Leukoc Biol (2022) 112(4):745–58. doi: 10.1002/JLB.5A0721-396RR 35258858

[15] Bozorgmehr N Hnatiuk M Peters AC Elahi S. Depletion of polyfunctional CD26(high)CD8(+) T cells repertoire in chronic lymphocytic leukemia. Exp Hematol Oncol (2023) 12(1):13. doi: 10.1186/s40164-023-00375-5 36707896 PMC9881277

[16] Sharma PK Wong EB Napier RJ Bishai WR Ndung'u T Kasprowicz VO. High expression of CD26 accurately identifies human bacteria-reactive MR1-restricted MAIT cells. Immunology (2015) 145(3):443–53. doi: 10.1111/imm.12461 PMC4479542 25752900

[17] Novak J Dobrovolny J Brozova J Novakova L Kozak T. Recovery of mucosal-associated invariant T cells after myeloablative chemotherapy and autologous peripheral blood stem cell transplantation. Clin Exp Med (2016) 16(4):529–37. doi: 10.1007/s10238-015-0384-z 26409838

[18] Solders M Erkers T Gorchs L Poiret T Remberger M Magalhaes I. Mucosal-associated invariant T cells display a poor reconstitution and altered phenotype after allogeneic hematopoietic stem cell transplantation. Front Immunol (2017) 8:1861. doi: 10.3389/fimmu.2017.01861 29312341 PMC5742569

[19] Bhattacharyya A Hanafi LA Sheih A Golob JL Srinivasan S Boeckh MJ. Graft-derived reconstitution of mucosal-associated invariant T cells after allogeneic hematopoietic cell transplantation. Biol Blood Marrow Transplant (2018) 24(2):242–51. doi: 10.1016/j.bbmt.2017.10.003 PMC5806215 29024803

[20] Kawaguchi K Umeda K Hiejima E Iwai A Mikami M Nodomi S. Influence of post-transplant mucosal-associated invariant T cell recovery on the development of acute graft-versus-host disease in allogeneic bone marrow transplantation. Int J Hematol (2018) 108(1):66–75. doi: 10.1007/s12185-018-2442-2 29582333

[21] Andrlova H Miltiadous O Kousa AI Dai A DeWolf S Violante S. MAIT and Vdelta2 unconventional T cells are supported by a diverse intestinal microbiome and correlate with favorable patient outcome after allogeneic HCT. Sci Transl Med (2022) 14(646):eabj2829. doi: 10.1126/scitranslmed.abj2829 35613281 PMC9893439

[22] Gao MG Hong Y Zhao XY Pan XA Sun YQ Kong J. The potential roles of mucosa-associated invariant T cells in the pathogenesis of gut graft-Versus-Host disease after hematopoietic stem cell transplantation. Front Immunol (2021) 12:720354. doi: 10.3389/fimmu.2021.720354 34539656 PMC8448388

[23] Wang Z Zhang S Zhang X Liu L Zhou L Shen Y. Mucosal-associated invariant T cells predict increased acute graft-versus-host-disease incidence in patients receiving allogeneic hematopoietic stem cell transplantation. Cancer Cell Int (2022) 22(1):297. doi: 10.1186/s12935-022-02703-x 36180885 PMC9526319

[24] Varelias A Bunting MD Ormerod KL Koyama M Olver SD Straube J. Recipient mucosal-associated invariant T cells control GVHD within the colon. J Clin Invest (2018) 128(5):1919–36. doi: 10.1172/JCI91646 PMC5919881 29629900

[25] Dusseaux M Martin E Serriari N Peguillet I Premel V Louis D. Human MAIT cells are xenobiotic-resistant, tissue-targeted, CD161hi IL-17-secreting T cells. Blood (2011) 117(4):1250–9. doi: 10.1182/blood-2010-08-303339 21084709

[26] Turtle CJ Swanson HM Fujii N Estey EH Riddell SR. A distinct subset of self-renewing human memory CD8+ T cells survives cytotoxic chemotherapy. Immunity (2009) 31(5):834–44. doi: 10.1016/j.immuni.2009.09.015 PMC2789980 19879163

[27] Robey RW Pluchino KM Hall MD Fojo AT Bates SE Gottesman MM. Revisiting the role of ABC transporters in multidrug-resistant cancer. Nat Rev Cancer (2018) 18(7):452–64. doi: 10.1038/s41568-018-0005-8 PMC6622180 29643473

[28] Leith CP Kopecky KJ Chen IM Eijdems L Slovak ML McConnell TS. Frequency and clinical significance of the expression of the multidrug resistance proteins MDR1/P-glycoprotein, MRP1, and LRP in acute myeloid leukemia: A southwest oncology group study. Blood (1999) 94(3):1086–99. 10419902

[29] Marie JP Zittoun R Sikic BI. Multidrug resistance (mdr1) gene expression in adult acute leukemias: Correlations with treatment outcome and *in vitro* drug sensitivity. Blood (1991) 78(3):586–92. doi: 10.1182/blood.V78.3.586.586 1859877

[30] Fergusson JR Ussher JE Kurioka A Klenerman P Walker LJ. High MDR-1 expression by MAIT cells confers resistance to cytotoxic but not immunosuppressive MDR-1 substrates. Clin Exp Immunol (2018) 194(2):180–91. doi: 10.1111/cei.13165 PMC6194332 30231297

[31] Keller AN Eckle SB Xu W Liu L Hughes VA Mak JY. Drugs and drug-like molecules can modulate the function of mucosal-associated invariant T cells. Nat Immunol (2017) 18(4):402–11. doi: 10.1038/ni.3679 28166217

[32] Jarvis EM Collings S Authier-Hall A Dasyam N Luey B Nacey J. Mucosal-associated invariant T (MAIT) cell dysfunction and PD-1 expression in prostate cancer: Implications for immunotherapy. Front Immunol (2021) 12:748741. doi: 10.3389/fimmu.2021.748741 34737749 PMC8560687

[33] Rodin W Sundstrom P Ahlmanner F Szeponik L Zajt KK Wettergren Y. Exhaustion in tumor-infiltrating mucosal-associated invariant T (MAIT) cells from colon cancer patients. Cancer Immunol Immunother (2021) 70(12):3461–75. doi: 10.1007/s00262-021-02939-y PMC8571139 33885944

[34] De Biasi S Gibellini L Lo Tartaro D Puccio S Rabacchi C Mazza EMC. Circulating mucosal-associated invariant T cells identify patients responding to anti-PD-1 therapy. Nat Commun (2021) 12(1):1669. doi: 10.1038/s41467-021-21928-4 33723257 PMC7961017

[35] Ruf B Catania VV Wabitsch S Ma C Diggs LP Zhang Q. Activating mucosal-associated invariant T cells induces a broad antitumor response. Cancer Immunol Res (2021) 9(9):1024–34. doi: 10.1158/2326-6066.CIR-20-0925 PMC8416791 34193462

[36] Zimmer CL Filipovic I Cornillet M O'Rourke CJ Berglin L Jansson H. Mucosal-associated invariant T-cell tumor infiltration predicts long-term survival in cholangiocarcinoma. Hepatology (2022) 75(5):1154–68. doi: 10.1002/hep.32222 34719787

[37] Lepore M Kalinichenko A Calogero S Kumar P Paleja B Schmaler M. Functionally diverse human T cells recognize non-microbial antigens presented by MR1. Elife (2017) 6. doi: 10.7554/eLife.24476 PMC5459576 28518056

[38] Crowther MD Dolton G Legut M Caillaud ME Lloyd A Attaf M. Genome-wide CRISPR-Cas9 screening reveals ubiquitous T cell cancer targeting *via* the monomorphic MHC class I-related protein MR1. Nat Immunol (2020) 21(2):178–85. doi: 10.1038/s41590-019-0578-8 PMC6983325 31959982

[39] Garrido F Aptsiauri N Doorduijn EM Garcia Lora AM van Hall T. The urgent need to recover MHC class I in cancers for effective immunotherapy. Curr Opin Immunol (2016) 39:44–51. doi: 10.1016/j.coi.2015.12.007 26796069 PMC5138279

[40] Cappell KM Kochenderfer JN. A comparison of chimeric antigen receptors containing CD28 versus 4-1BB costimulatory domains. Nat Rev Clin Oncol (2021) 18(11):715–27. doi: 10.1038/s41571-021-00530-z 34230645

[41] Bailey SR Berger TR Graham C Larson RC Maus MV. Four challenges to CAR T cells breaking the glass ceiling. Eur J Immunol (2022) 31:e2250039. doi: 10.1002/eji.202250039 36585889

[42] Bohineust A Tourret M Derivry L Caillat-Zucman S. Mucosal-associated invariant T (MAIT) cells, a new source of universal immune cells for chimeric antigen receptor (CAR)-cell therapy. Bull Cancer (2021) 108(10S):S92–S5. doi: 10.1016/j.bulcan.2021.07.003 34920812

[43] Tourret M Talvard-Balland N Lambert M Ben Youssef G Chevalier MF Bohineust A. Human MAIT cells are devoid of alloreactive potential: prompting their use as universal cells for adoptive immune therapy. J Immunother Cancer (2021) 9(10). doi: 10.1136/jitc-2021-003123 PMC8496386 34615705

[44] Dogan M Karhan E Kozhaya L Placek L Chen X Yigit M. Engineering human MAIT cells with chimeric antigen receptors for cancer immunotherapy. J Immunol (2022) 209(8):1523–31. doi: 10.4049/jimmunol.2100856 36165183

[45] Nianias A Themeli M. Induced pluripotent stem cell (iPSC)-derived lymphocytes for adoptive cell immunotherapy: Recent advances and challenges. Curr Hematol Malig Rep (2019) 14(4):261–8. doi: 10.1007/s11899-019-00528-6 PMC6647376 31243643

[46] Sugimoto C Murakami Y Ishii E Fujita H Wakao H. Reprogramming and redifferentiation of mucosal-associated invariant T cells reveal tumor inhibitory activity. Elife (2022) 11. doi: 10.7554/eLife.70848 PMC8983048 35379387

[47] Wakao H Yoshikiyo K Koshimizu U Furukawa T Enomoto K Matsunaga T. Expansion of functional human mucosal-associated invariant T cells *via* reprogramming to pluripotency and redifferentiation. Cell Stem Cell (2013) 12(5):546–58. doi: 10.1016/j.stem.2013.03.001 23523177

[48] Tian Z Liu M Zhang Y Wang X. Bispecific T cell engagers: An emerging therapy for management of hematologic malignancies. J Hematol Oncol (2021) 14(1):75. doi: 10.1186/s13045-021-01084-4 33941237 PMC8091790

[49] Yang R He Q Zhang J Yan Y Shi J Zhou P. The redirected killing of PD-L1 positive tumor cells by the expanded mucosa-associated invariant T (MAIT) cells is mediated with a bispecific antibody targeting TCR Valpha7.2 and PD-L1. Biochem Biophys Res Commun (2023) 644:1–7. doi: 10.1016/j.bbrc.2023.01.006 36621147

[50] Berraondo P Sanmamed MF Ochoa MC Etxeberria I Aznar MA Perez-Gracia JL. Cytokines in clinical cancer immunotherapy. Br J Cancer (2019) 120(1):6–15. doi: 10.1038/s41416-018-0328-y 30413827 PMC6325155

[51] Lin MJ Svensson-Arvelund J Lubitz GS Marabelle A Melero I Brown BD. Cancer vaccines: The next immunotherapy frontier. Nat Cancer (2022) 3(8):911–26. doi: 10.1038/s43018-022-00418-6 35999309

[52] Dey RJ Dey B Harriff M Canfield ET Lewinsohn DM Bishai WR. Augmentation of the riboflavin-biosynthetic pathway enhances mucosa-associated invariant T (MAIT) cell activation and diminishes mycobacterium tuberculosis virulence. mBio (2022) 13(1):e0386521. doi: 10.1128/mbio.03865-21 PMC8844931 35164552

[53] Jensen O Trivedi S Li K Aube J Hale JS Ryan ET. Use of a MAIT-activating ligand, 5-OP-RU, as a mucosal adjuvant in a murine model of vibrio cholerae O1 vaccination. Pathog Immun (2022) 7(1):122–44. doi: 10.20411/pai.v7i1.525 PMC9438945 36072570

[54] Provine NM Amini A Garner LC Spencer AJ Dold C Hutchings C. MAIT cell activation augments adenovirus vector vaccine immunogenicity. Science (2021) 371(6528):521–6. doi: 10.1126/science.aax8819 PMC7610941 33510029

[55] Grasso C Field CS Tang CW Ferguson PM JC B Anderson RJ. Vaccines adjuvanted with an NKT cell agonist induce effective T-cell responses in models of CNS lymphoma. Immunotherapy (2020) 12(6):395–406. doi: 10.2217/imt-2019-0134 32316797

[56] Mattarollo SR West AC Steegh K Duret H Paget C Martin B. NKT cell adjuvant-based tumor vaccine for treatment of myc oncogene-driven mouse b-cell lymphoma. Blood (2012) 120(15):3019–29. doi: 10.1182/blood-2012-04-426643 PMC3557399 22932803

[57] Varghese B Lynch L Vriend LE Draganov D Clark JM Kissick HT. Invariant NKT cell-augmented GM-CSF-secreting tumor vaccine is effective in advanced prostate cancer model. Cancer Immunol Immunother (2022) 71(12):2943–55. doi: 10.1007/s00262-022-03210-8 PMC10992623 35523889

[58] Leeansyah E Ganesh A Quigley MF Sonnerborg A Andersson J Hunt PW. Activation, exhaustion, and persistent decline of the antimicrobial MR1-restricted MAIT-cell population in chronic HIV-1 infection. Blood (2013) 121(7):1124–35. doi: 10.1182/blood-2012-07-445429 PMC3575756 23243281

[59] Saeidi A Tien Tien VL Al-Batran R Al-Darraji HA Tan HY Yong YK. Attrition of TCR Valpha7.2+ CD161++ MAIT cells in HIV-tuberculosis co-infection is associated with elevated levels of PD-1 expression. PloS One (2015) 10(4):e0124659. doi: 10.1371/journal.pone.0124659 25894562 PMC4403924

[60] Barathan M Mohamed R Vadivelu J Chang LY Saeidi A Yong YK. Peripheral loss of CD8(+) CD161(++) TCRValpha7.2(+) mucosal-associated invariant T cells in chronic hepatitis c virus-infected patients. Eur J Clin Invest (2016) 46(2):170–80. doi: 10.1111/eci.12581 26681320

[61] Dias J Hengst J Parrot T Leeansyah E Lunemann S Malone DFG. Chronic hepatitis delta virus infection leads to functional impairment and severe loss of MAIT cells. J Hepatol (2019) 71(2):301–12. doi: 10.1016/j.jhep.2019.04.009 PMC6642010 31100314

[62] Huang W He W Shi X Ye Q He X Dou L. Mucosal-associated invariant T-cells are severely reduced and exhausted in humans with chronic HBV infection. J Viral Hepat (2020) 27(11):1096–107. doi: 10.1111/jvh.13341 32510704

[63] Yong YK Saeidi A Tan HY Rosmawati M Enstrom PF Batran RA. Hyper-expression of PD-1 is associated with the levels of exhausted and dysfunctional phenotypes of circulating CD161(++)TCR iValpha7.2(+) mucosal-associated invariant T cells in chronic hepatitis b virus infection. Front Immunol (2018) 9:472. doi: 10.3389/fimmu.2018.00472 29616020 PMC5868455

[64] Nel I Beaudoin L Gouda Z Rousseau C Soulard P Rouland M. MAIT cell alterations in adults with recent-onset and long-term type 1 diabetes. Diabetologia (2021) 64(10):2306–21. doi: 10.1007/s00125-021-05527-y PMC8336671 34350463

[65] Bottcher K Rombouts K Saffioti F Roccarina D Rosselli M Hall A. MAIT cells are chronically activated in patients with autoimmune liver disease and promote profibrogenic hepatic stellate cell activation. Hepatology (2018) 68(1):172–86. doi: 10.1002/hep.29782 29328499

[66] Li S Simoni Y Becht E Loh CY Li N Lachance D. Human tumor-infiltrating MAIT cells display hallmarks of bacterial antigen recognition in colorectal cancer. Cell Rep Med (2020) 1(3):100039. doi: 10.1016/j.xcrm.2020.100039 33205061 PMC7659584

[67] Hengst J Strunz B Deterding K Ljunggren HG Leeansyah E Manns MP. Nonreversible MAIT cell-dysfunction in chronic hepatitis c virus infection despite successful interferon-free therapy. Eur J Immunol (2016) 46(9):2204–10. doi: 10.1002/eji.201646447 27296288

[68] Berkson JD Slichter CK DeBerg HA Delaney MA Woodward-Davis AS Maurice NJ. Inflammatory cytokines induce sustained CTLA-4 cell surface expression on human MAIT cells. Immunohorizons (2020) 4(1):14–22. doi: 10.4049/immunohorizons.1900061 31974109 PMC7357853

[69] Kelly J Minoda Y Meredith T Cameron G Philipp MS Pellicci DG. Chronically stimulated human MAIT cells are unexpectedly potent IL-13 producers. Immunol Cell Biol (2019) 97(8):689–99. doi: 10.1111/imcb.12281 PMC6790710 31323167

[70] Kurioka A Ussher JE Cosgrove C Clough C Fergusson JR Smith K. MAIT cells are licensed through granzyme exchange to kill bacterially sensitized targets. Mucosal Immunol (2015) 8(2):429–40. doi: 10.1038/mi.2014.81 PMC4288950 25269706

[71] Turtle CJ Delrow J Joslyn RC Swanson HM Basom R Tabellini L. Innate signals overcome acquired TCR signaling pathway regulation and govern the fate of human CD161(hi) CD8alpha(+) semi-invariant T cells. Blood (2011) 118(10):2752–62. doi: 10.1182/blood-2011-02-334698 PMC3172793 21791427

[72] Chen Z Wang H D'Souza C Sun S Kostenko L Eckle SB. Mucosal-associated invariant T-cell activation and accumulation after *in vivo* infection depends on microbial riboflavin synthesis and co-stimulatory signals. Mucosal Immunol (2017) 10(1):58–68. doi: 10.1038/mi.2016.39 27143301

[73] Pavlovic M Gross C Chili C Secher T Treiner E. MAIT cells display a specific response to type 1 IFN underlying the adjuvant effect of TLR7/8 ligands. Front Immunol (2020) 11:2097. doi: 10.3389/fimmu.2020.02097 33013883 PMC7509539

[74] Lamichhane R Schneider M de la Harpe SM Harrop TWR Hannaway RF Dearden PK. TCR- or cytokine-activated CD8(+) mucosal-associated invariant T cells are rapid polyfunctional effectors that can coordinate immune responses. Cell Rep (2019) 28(12):3061–76 e5. doi: 10.1016/j.celrep.2019.08.054 31533031

[75] Hinks TSC Marchi E Jabeen M Olshansky M Kurioka A Pediongco TJ. Activation and *In vivo* evolution of the MAIT cell transcriptome in mice and humans reveals tissue repair functionality. Cell Rep (2019) 28(12):3249–62 e5. doi: 10.1016/j.celrep.2019.07.039 31533045 PMC6859474

[76] Leng T Akther HD Hackstein CP Powell K King T Friedrich M. TCR and inflammatory signals tune human MAIT cells to exert specific tissue repair and effector functions. Cell Rep (2019) 28(12):3077–91 e5. doi: 10.1016/j.celrep.2019.08.050 31533032 PMC6899450

[77] Constantinides MG Link VM Tamoutounour S Wong AC Perez-Chaparro PJ Han SJ. MAIT cells are imprinted by the microbiota in early life and promote tissue repair. Science (2019) 366(6464):eaax6624. doi: 10.1126/science.aax6624 31649166 PMC7603427

[78] du Halgouet A Darbois A Alkobtawi M Mestdagh M Alphonse A Premel V. Role of MR1-driven signals and amphiregulin on the recruitment and repair function of MAIT cells during skin wound healing. Immunity (2023) 56(1):78–92 e6. doi: 10.1016/j.immuni.2022.12.004 36630919 PMC9839364

[79] Ben Youssef G Tourret M Salou M Ghazarian L Houdouin V Mondot S. Ontogeny of human mucosal-associated invariant T cells and related T cell subsets. J Exp Med (2018) 215(2):459–79. doi: 10.1084/jem.20171739 PMC5789419 29339446

[80] Lee OJ Cho YN Kee SJ Kim MJ Jin HM Lee SJ. Circulating mucosal-associated invariant T cell levels and their cytokine levels in healthy adults. Exp Gerontol (2014) 49:47–54. doi: 10.1016/j.exger.2013.11.003 24269212

[81] Novak J Dobrovolny J Novakova L Kozak T. The decrease in number and change in phenotype of mucosal-associated invariant T cells in the elderly and differences in men and women of reproductive age. Scand J Immunol (2014) 80(4):271–5. doi: 10.1111/sji.12193 24846411

[82] van der Geest KSM Kroesen BJ Horst G Abdulahad WH Brouwer E Boots AMH. Impact of aging on the frequency, phenotype, and function of CD161-expressing T cells. Front Immunol (2018) 9:752. doi: 10.3389/fimmu.2018.00752 29725326 PMC5917671

[83] Di Rosa F Gebhardt T. Bone marrow T cells and the integrated functions of recirculating and tissue-resident memory T cells. Front Immunol (2016) 7:51. doi: 10.3389/fimmu.2016.00051 26909081 PMC4754413

[84] Noonan KA Huff CA Davis J Lemas MV Fiorino S Bitzan J. Adoptive transfer of activated marrow-infiltrating lymphocytes induces measurable antitumor immunity in the bone marrow in multiple myeloma. Sci Transl Med (2015) 7(288):288ra78. doi: 10.1126/scitranslmed.aaa7014 PMC4634889 25995224

[85] Mazo IB Honczarenko M Leung H Cavanagh LL Bonasio R Weninger W. Bone marrow is a major reservoir and site of recruitment for central memory CD8+ T cells. Immunity (2005) 22(2):259–70. doi: 10.1016/j.immuni.2005.01.008 15723813

[86] Willing A Leach OA Ufer F Attfield KE Steinbach K Kursawe N. CD8(+) MAIT cells infiltrate into the CNS and alterations in their blood frequencies correlate with IL-18 serum levels in multiple sclerosis. Eur J Immunol (2014) 44(10):3119–28. doi: 10.1002/eji.201344160 25043505

[87] Goedhart M Gessel S van der Voort R Slot E Lucas B Gielen E. CXCR4, but not CXCR3, drives CD8(+) T-cell entry into and migration through the murine bone marrow. Eur J Immunol (2019) 49(4):576–89. doi: 10.1002/eji.201747438 30707456

[88] Dias J Leeansyah E Sandberg JK. Multiple layers of heterogeneity and subset diversity in human MAIT cell responses to distinct microorganisms and to innate cytokines. Proc Natl Acad Sci USA (2017) 114(27):E5434–E43. doi: 10.1073/pnas.1705759114 PMC5502643 28630305

[89] Chen Z Liu S He C Sun J Wang L Chen H. CXCL12-CXCR4-Mediated chemotaxis supports accumulation of mucosal-associated invariant T cells into the liver of patients with PBC. Front Immunol (2021) 12:578548. doi: 10.3389/fimmu.2021.578548 33815355 PMC8017208

[90] Tettamanti S Pievani A Biondi A Dotti G Serafini M. Catch me if you can: how AML and its niche escape immunotherapy. Leukemia (2022) 36(1):13–22. doi: 10.1038/s41375-021-01350-x 34302116 PMC8727297

[91] Diaz-Tejedor A Lorenzo-Mohamed M Puig N Garcia-Sanz R Mateos MV Garayoa M. Immune system alterations in multiple myeloma: Molecular mechanisms and therapeutic strategies to reverse immunosuppression. Cancers (Basel) (2021) 13(6). doi: 10.3390/cancers13061353 PMC8002455 33802806

